# Incontinence of Urine

**Published:** 1851-08

**Authors:** 


*Incontinence of Urine.*—M. Demaux has published two cases in the *Gazette des Hôspitaux,* January, 1851, one of a young man of twenty, the other referring to a girl of eighteen years, in which he succeeded in arresting the uncomfortable flow of urine at night by cauterizing the neck of the bladder with nitrate of silver, using Lallemand's "porte caustique" for the purpose. Various remedies had been tried in both cases, but they had all failed; cauterization, however, removed the symptoms in a very short time.—*Lon. Lancet.*

